# Global Disparities in the Regulation of PFASs: The Risk of Shifting the PFAS Pollution Burden to Developing Countries

**DOI:** 10.1021/acs.est.5c14777

**Published:** 2026-03-10

**Authors:** Brij Mohan Sharma, Ian T. Cousins, Hans Peter H. Arp, Martin Scheringer

**Affiliations:** † RECETOX, Faculty of Science, Masaryk University, Kotlarska 2, Brno 60200, Czech Republic; ‡ Institute of Biogeochemistry and Pollutant Dynamics (IBP), ETH Zürich, Zürich 8092, Switzerland; § Department of Environmental Science, Stockholm University, 106 91 Stockholm, Sweden; ∥ Norwegian Geotechnical Institute, 0484 Oslo, Norway; ⊥ Norwegian University of Science and Technology, 7034 Trondheim, Norway

**Keywords:** PFAS pollution, PFAS restrictions, regulatory disparities, developing countries, cocreation

## Abstract

The environmental health challenges of per- and polyfluoroalkyl substances (PFASs) are well-documented in developed countries, where serious efforts are underway to implement stricter regulations to lower PFAS emissions. However, in developing countries where PFASs have been detected at levels similar to those in developed countries, there is a lack of comparable research or efforts on addressing PFAS pollution. These gaps also apply to many other industrial chemicals and are underpinned by imbalances in chemical regulation between developed and developing countries. These imbalances are likely to create multifaceted global challenges, including the illegal use and trade of PFASs and their products, the relocation of PFAS-based industries, and the global recirculation of PFAS pollution. These challenges can exacerbate pressure on developing countries already grappling with other critical environmental issues. In this Perspective, we explore these challenges arising from global disparities in the regulation of PFASs and other chemicals, along with their repercussions. We propose solutions to bridge the regulatory gaps, including broad, worldwide PFAS bans and regulations, increased funding for PFAS monitoring and emissions reduction, and joint initiatives with developed countries. These efforts would ensure that PFAS management extends beyond the developed world to countries with high economic aspirations and limited resources to address chemical pollution.

## Graphical abstract

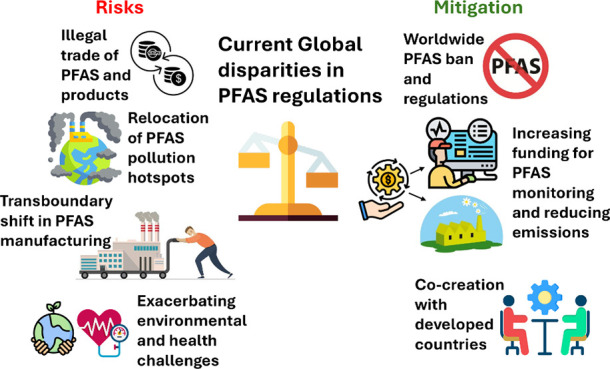

## Introduction

In recent years, per- and polyfluoroalkyl substances (PFASs) have received growing global attention from researchers, policymakers, and civil society. PFASs are a large group of synthetic chemicals defined as fluorinated substances that contain at least one fully fluorinated methyl or methylene carbon atom (without any H/Cl/Br/I atom attached to it), i.e. with a few noted exceptions, any chemical with at least a perfluorinated methyl group (−CF_3_) or a perfluorinated methylene group (−CF_2_−) is a PFAS.^1,2^ They have a wide range of applications, including in TULAC (textiles, upholstery, leather, apparel, and carpets), firefighting foams, healthcare, biotechnology, building and construction, aviation, electronics industry, the energy sector, food production, packaging, mining, etc.^3^ One of the key and concerning characteristics of these substances is their extreme resistance to degradation, i.e. their high persistence in the environment, which in many cases may last thousands of years.^4,5^ Many PFASs have been found ubiquitously in the environment, wildlife, and human populations across the globe, often exceeding safe levels.^6,7^ Exposure to some of the well-studied PFASs such as perfluorooctanoic acid (PFOA) and perfluorooctanesulfonate (PFOS) may result in serious health consequences including various types of cancers, thyroid diseases, decreased immunity and endocrine disruption, whereas the health risks of many other PFASs are still unknown and being studied.^8^

PFASs are an exemplary case of serious chemical management challenges to policymakers due to the increasing number of chemicals entering the global chemicals market, many of which lack sufficient hazard data.^9,10^ Due to their vast number, widespread uses, and the availability of alternatives and toxicity information for only a subset of PFASs, managing them presents a unique challenge of a scale and significance never before faced in global chemicals management.^11,12^ Given the severity and extent of the PFAS problem, coordinated international regulatory efforts on their production and emission reduction and on waste management are required for effectively protecting human health and the environment. This would require increasing the number of international instruments for managing chemicals. Currently, the Stockholm Convention on Persistent Organic Pollutants (POPs) is the only existing multilateral environmental agreement (MEA) that bans/restricts (with certain exemptions) a small subset of PFASs.^13^ Further in this direction, progress has been made in selected regions and countries, including the European Union (EU), the US, the UK, Australia, Canada, and to an extent, in Japan, South Korea, and China, where comprehensive PFAS-specific regulations have been framed (or proposed). These often include bans and restrictions on the use and production of larger groups of PFASs and PFAS-containing products (in some cases extending to all PFASs, except for a few essential uses) as compared to the contemporary regulations, particularly in developing countries, dealing with PFASs (see Table 1 for a summary of regulations and policies, and Figure 1 for the numbers of PFASs included in various regulatory databases).^14^ Apart from these selected regions and countries, many countries around the world have obligations under the Stockholm Convention, which includes a small subset of PFASs in its list of POPs. As parties to the convention, these countries are required to update their National Implementation Plans (NIPs), which document the national implementation of restrictions on this small subset of PFASs. For example, NIPs based on the Conference of the Parties (COP4)-amendments of the Stockholm Convention show regulatory activity addressing PFOS (its salts and perfluorooctane sulfonyl fluoride (PFOSF)) in certain developing countries including Suriname, Kenya, Argentina, Indonesia, etc.^15,16^ Nevertheless, implementation of these regulatory mechanisms remains weak or outdated in many developing regions, leading to no systematic control over widespread PFAS pollution.^17,18^ This fragmented regulatory landscape, where more comprehensive frameworks exist in only a handful of countries and a single multilateral environmental agreement covers only a small number of PFASs, leads to uneven implementation across regions. This will allow PFAS risks to persist in much of the world, as well as eventually spread from areas with less regulation to more regulation.^14,19^

**Table 1:** A Summary of Regulations and Policies on PFAS Management Across Different Countries and Regions Where the Regulatory Scope Extends beyond PFASs Listed under the Stockholm Convention^a^

European Union
•Persistent Organic Pollutants (POPs) Regulation (EU 2019/1021): Bans perfluorooctanesulfonic acid (PFOS) and its salts; perfluorooctanesulfonylfluoride (PFOS-F); perfluorooctanoic acid (PFOA), perfluorohexanesulfonic acid (PFHxS), its salts, and related compounds. It legally enforces concentration limits for unintentional trace contaminants in products and wastes and sometimes applies stricter phase-out schedules or fewer exemptions than found in the Stockholm Convention.
•Registration, Evaluation, Authorisation, and Restriction of Chemicals (REACH) (EC 1907/2006): A primary step toward a potential future restriction or authorization of PFASs through inclusions in the candidate list of Substances of Very High Concern (SVHC), including 2,3,3,3-tetrafluoro-2-(heptafluoropropoxy)propionic acid, its salts and acyl halides (HFPO–DA, also known as GenX chemicals); perfluorobutanesulfonic acid (PFBS) and its salts; perfluoroheptanoic acid (PFHpA) and its salts; and perfluorocarboxylic acids (C9–C14 PFCAs) and their salts.
•The REACH restrictions on PFHxA, its salts and PFHxA-related substances (Commission Regulation (EU) 2024/2462).
•The Classification, Labelling and Packaging (CLP) Regulation (EC 1272/2008): Mandates classification, labeling, and communication of the hazards of PFASs in the EU market, for which a harmonized classification exists for PFOA; including ammonium pentadecafluorooctanoate (APFO); perfluorononan-1-oic acid (PFNA) and its sodium and ammonium salts; nonadecafluorodecanoic acid (PFDA) and its sodium and ammonium salts; and PFHpA.
•Drinking Water Directive (DWD) (EU 2020/2184): Establishes permissible limits for PFAS Total (all PFASs) and the sum of 20 target PFASs that are specified in the regulation (C4–C13 PFCAs and C4–C13 PFSAs).
•Prior Informed Consent (PIC) Regulations (EU 649/2012): The EU regulation implementing the UN Rotterdam Convention, which governs the environmentally sound movement and use of hazardous chemicals including POP–PFASs. The scope of the PIC Regulation goes beyond the Rotterdam Convention at the EU level, as it also imposes a ban on the export of POP–PFASs to both EU and non-EU countries, thereby going a step further than the Convention’s “prior informed consent” procedure.
•Urban Wastewater Treatment Directive (EU 2024/3019): Includes a monitoring requirement for specified PFASs.
•Restriction on PFASs in firefighting foams (Commission Regulation (EU) 2025/1988) (amendment to Annex XVII of the REACH Regulation).
•Member state specific regulations: Certain EEA members, e.g. France, Belgium, Denmark, Sweden and Norway have imposed bans on all or specific PFASs in consumer products such as clothing, footwear, cosmetics, and ski wax.
•The Environmental Quality Standards Directive, the Groundwater Directive, and the Water Framework Directive have added Environmental Quality standards for 25 PFASs, including trifluoroacetic acid (TFA).
*Emerging regulations*
•Soil Monitoring and Resilience Directive (EU 2025/2360): Includes a monitoring requirement for selected PFASs, based on capacities of national laboratories.
•Broad PFAS Restriction Proposal: Mandates the phased elimination, use reduction or emission reduction of all PFASs for industrial uses under the REACH regulation, which would exclude active substances in certain plant protection, biocidal, and medicinal products.

North America
USA
•US EPA TSCA: Bans the use of long-chain PFASs in food-contact materials.
•The National Defense Authorization Act (NDAA): Directs the inclusion of 172 PFASs in the Toxic Release Inventory, even though PFASs are not yet listed as hazardous substances under a federal program.
•NDAA: Enacts a series of mandates to transition from aqueous film-forming foam (AFFF) to fluorine-free foam (F3) alternatives.
•US EPA (Drinking Water Guidance): Establishes a series of Regional Screening Levels (RSLs) for PFOA, PFOS, PFNA, PFBS, PFHxS, and HFPO–DA in groundwater. These RSLs are not promulgated rules and are, therefore, not legally enforceable limits under the statute.
*Emerging regulations*
•The Safe Drinking Water Act (proposed rule): Establishes national primary drinking water standards for PFOA and PFOS, and sets limits for PFOA, PFOS, PFNA, PFHxS, PFBS, and HFPO–DA using a combined Hazard Index of 1.0.
•US EPA (guidance to States): Recommends permit restrictions and monitoring requirements to limit PFAS discharges from industrial sources to waterways.
•Comprehensive Environmental Response, Compensation, and Liability Act (CERCLA): Designates PFOA and PFOS as hazardous substances under CERCLA.
Canada
•Canadian Environmental Protection Act (CEPA): Bans manufacturing, use, sale, and import of PFOS, PFOA, long-chain (C9–C20) PFCAs, and their collective salts and precursors.
•CEPA (Firefighting Foam Use): Bans the use of AFFFs, with a few exemptions for overseas military operational and to support transition to F3.
•Drinking water standards: Sets no promulgated national drinking water standards for PFASs nationally; Alberta, however, has established maximum allowable concentrations of 200 ppt for PFOA and 600 ppt for PFOS in the ambient water quality guideline for drinking water resources.

Asia-Pacific
Australia
•Government Position Statement: Use of PFASs should be limited to the greatest extent practicable and sets objectives for phasing-out the uses of PFASs of concern in Australia.
•Industrial Chemicals Act 2019 (ICAct): Requires importers and manufacturers of PFASs to comply with the regulatory obligations.
•Australian Industrial Chemicals Introduction Scheme (AICIS): Enforces import and export controls on PFOS and specified PFOS precursors subject to the prior informed consent procedure (PIC) under the Rotterdam Convention.
•A ban on the import, manufacture, export, and intentional use of PFOS, PFOA, PFHxS, including their salts, isomers, and precursors, under the Industrial Chemicals Environmental Management Standard (IChEMS), effective from 1 July 2025, and restricting more than 500 PFASs.
•State Regulations: South Australia, Queensland, and New South Wales restrict the use of certain PFASs in firefighting foams; South Australia was the first state to ban all PFAS-containing firefighting foams.
•PFAS National Environmental Management Plan (NEMP): Promotes flexible implementation of best practice and provides practical guidance for the investigation and management of PFAS contamination, including waste management, storage and disposal.
New Zealand
•Firefighting Foam restrictions: Restricts the use of AFFFs as of 21 Dec 2022.
•Use of firefighting foams containing PFASs in uncontained systems is prohibited.
•Plans to implement a complete ban on PFAS-containing firefighting foams starting from 3 Dec 2025.
Japan
•Chemical Substances Control Law (CSCL): Classifies 138 PFASs as class I Specified Chemical Substances and prohibits their manufacture, import, and use.
China
•Ministry of Ecology and Environment (priority list): Includes certain PFASs in its list of priority-controlled chemicals, requiring strict reporting and monitoring.
•Control measures for 363 PFASs including PFOA, its salts and PFOA-related compounds.
South Korea
•The South Korean Act on Registration and Evaluation of Chemical Substances (K-REACH): Focuses on the registration and evaluation of PFASs and imposes restrictions on their use.
•K-REACH: Designates some PFASs as “Toxic Substances” or “Substances subject to Intensive Control”.
•POPs Control Act: Restricts or bans PFOA, PFOS, and PFHxS. In addition to these three PFASs, their POP measurement network includes five PFASs namely PFBA, PFBS, PFHxA, PFNA, and PFDA.

a The table is synthesized based on the work by Thomas et al. and Yu et al.^14,19^ .

**Figure 1:**
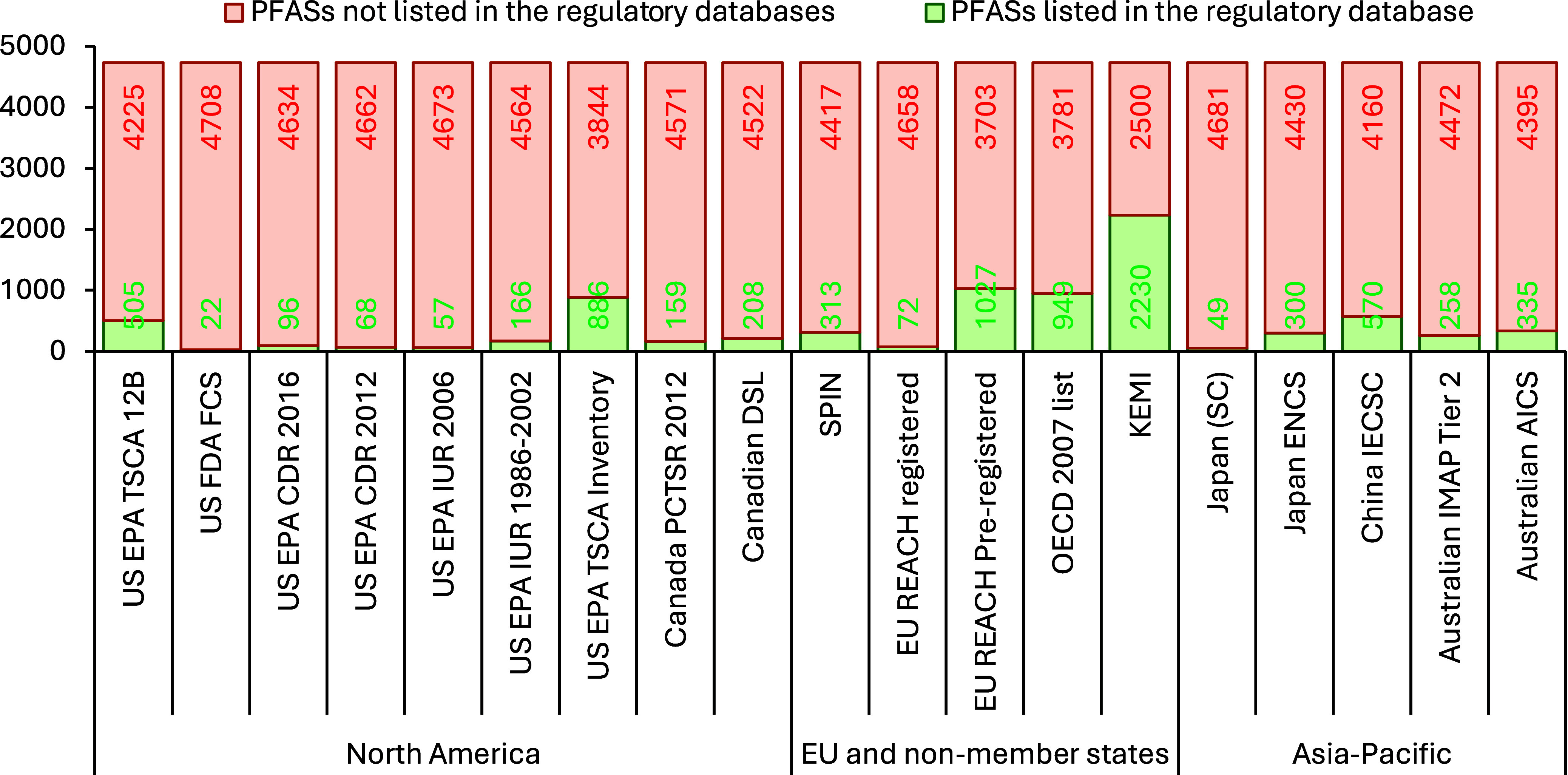
Number of PFASs listed (and remaining to be listed) out of an estimated 4730 different PFASs that could be in the global market, across the regulatory databases of different countries in North America, the EU and non-member states, and the Asia-Pacific region. This figure is adapted from the work of Yu et al.^19^

The most ambitious of these efforts in developed regions is the EU’s broad restriction proposal for all PFASs applicable under the REACH regulation, which excludes active ingredients in certain plant protection, biocidal, and medicinal products.^20,21^ The required transition to alternatives may be particularly difficult, at least in the immediate future, for certain sectors where safe, sustainable, and economic PFAS alternatives are still being explored.^22^ The difficulty in phasing out PFASs in certain sectors stems from several key factors: (i) currently PFASs (with known and unknown toxicity) are an integral part of some critically important sectors such as clean energy and health care.^3^ Importantly, these sectors contribute to some of the UN Sustainable Development Goals (SDGs) such as “affordable and clean energy” and “good health and well-being”, making immediate replacement of PFASs difficult yet not impossible; (ii) there are effective, safe, and sustainable alternatives to an increasing number of PFASs but not yet for all,^23^ and (iii) the timeline for developing and widely releasing PFAS-free safe and sustainable alternatives to replace all current uses in the market remains uncertain.^24^ This challenge is also reflected in the EU’s PFASs restriction proposal, which allows for a derogation period of up to 12 years, where technically and economically feasible alternatives are not yet available in the market.^21^ To further accelerate the search for PFAS alternatives and the phase-out of current PFAS use and production, both the European Commission and scientists have called for inclusion of the ‘essential use’ concept in the EU and global chemicals regulation, to more rapidly phase out PFASs for uses when their technical function is not strictly needed or when safer alternatives can replace PFASs with an adequate level of performance.^25,26^

While the EU’s restriction proposal (along with other countries’ progressive efforts in PFAS management) represents a critical step forward, these challenges highlight that even in regions with strong regulatory ambitions, the path to phasing out PFASs is complex. Here, we introduce a new perspective to these existing challenges on global PFAS management, highlighting that regulatory disparities between developed and developing countries in managing the production, use, and trade of PFASs (and products containing them) will further exacerbate global PFAS pollution and its associated environmental and health burdens. Hereafter we highlight and discuss priority issues that are expected to emerge from the global disparities in PFAS regulations and chemicals management actions, and ways to address them.

## Issues Emerging from Fragmented International PFAS and Chemicals Regulation

### Illegal Trade (And Production) of PFASs and PFAS-Containing Products

The recent PFAS ban proposals in the EU and the US, including restrictions on certain consumer and industrial products containing PFASs in the states of New York, California, etc., as well as in a few other developed countries, are estimated to cost industries billions of USD in transitioning away from PFASs. These costs include the expenses associated with renewing their processes, retooling equipment, readjusting supply chains and investing in research and development to create safe and sustainable PFAS-free alternatives.^27^ However, it is important to recognize that the benefits of banning PFASs will even outweigh the industrial costs, as these measures are expected to lead to significant environmental and public health protection from the harmful effects of PFAS exposure and further reduce costs associated with future environmental cleanups and disease burdens.^28,29^ While large industries typically seek to maximize their immediate profits, weak or absent PFAS regulations in developing countries offer them a safe haven for offloading existing PFAS stocks and PFAS-containing consumer and industrial products that are discarded or phased out in the markets with stricter regulations—a typical example of the “race to the bottom” concept.^30^ Moreover, the EU’s PFAS restriction proposal allows industries up to about 12 years to transition to PFAS-free processes and products. Meanwhile, only a few countries outside the EU, such as the US, UK, Canada, Australia, Japan, South Korea, and China, have adopted regulatory measures targeting a larger group of PFASs beyond the Stockholm Convention (Table 1), whereas the majority of countries worldwide have not yet proposed or adopted such PFAS restriction plans and timelines to phase them out. Information on PFAS manufacturing, uses, and regulations in developing countries is limited. This is often due to inadequate regulatory requirements for declaring PFAS stocks by producers and consumers (at the manufacturing levels), an absence of database platforms for chemicals in general, as well as overlapping mandates and responsibilities among national regulatory authorities concerning overall chemical management. Such regulatory inconsistency creates opportunities for industries to relocate and continue using PFAS products and processes in countries with weaker or absent PFAS regulations and restriction timelines. While direct data on PFAS (and products containing them) trade are scarce, broader historical patterns in the movement of prohibited hazardous chemicals and products due to disparities in chemical regulations between developed and developing countries can provide a relevant risk context here. For instance, a recent investigation found that agrochemical companies in the EU and UK exported tens of thousands of tonnes of pesticides that were banned in Europe to low- and middle-income countries around the world.^31^ Another assessment of the effectiveness of the Rotterdam Convention (to regulate the transboundary movements of hazardous chemicals) showed that from 2004 to 2019, about 27.5 million tonnes of 46 listed hazardous chemicals were traded illegally.^32^ Similarly, of the total global circulation of waste electronic and electrical equipment (WEEE), which is subject to the Basel Convention, 65% (3.3 billion kg annually) is uncontrolled transboundary movement from high-income to middle- and low-income countries. Most of this uncontrolled WEEE primarily originates in Europe and North America and is destined for countries in West Africa and Southeast Asia.^33–35^ While PFASs and products containing them have unique uses and trade dynamics (in addition to their uses in pesticides and in electronic equipment that becomes WEEE),^36,37^ these examples illustrate a broader pattern in which hazardous chemicals restricted in one region may continue to be traded to or produced in developing countries. Such precedents highlight potential risks and challenges in managing the global circulation of PFASs (if sporadically restricted and banned) and indicate the urgent need for improved data and regulatory transparency regarding PFAS management (including trade) in both developed and developing countries.

In addition to the illegal transboundary movement of banned chemicals, researchers under a globally coordinated monitoring program on ozone-depleting substances found solid scientific evidence of illegal production and use of these substances in developing countries, despite their prohibition under both the Montreal Protocol, and national regulations.^38,39^ Specifically, high emissions of trichlorofluoromethane (CFC-11), a substance banned under the Montreal Protocol, were detected, pointing to its illegal large-scale production and use in China.^40^ This finding validated an earlier published UN report that identified China as a hub of illegal trade, trafficking and production of chlorofluorocarbons.^41^ This example of illegal production and use and continued global pollution by highly persistent and harmful chemicals that are controlled under multilateral agreements underscores concerns relevant to PFASs, including relocation of their (illegal) production and use in less regulated markets once stricter regulations are imposed in developed countries. However, unlike CFCs, currently the international trade of most PFASs is not inherently illegal, as only a small subset of PFASs is listed under the Stockholm Convention and subject to global bans and restrictions. For the vast majority of PFASs, international trade remains permitted but increasingly regulated through reporting, labeling, and registration requirements such as those under the EU REACH or the US Toxic Substances Control Act (TSCA). The challenge, therefore, lies not only in illegal trade but also in the complexity of ensuring compliance, transparency, and consistent enforcement across jurisdictions. For a smaller group of PFASs listed under the Stockholm Convention, a global ban or restriction on their production, use, and trade is an important step forward; however, its effectiveness depends heavily on its timely ratification and enforcement at the domestic level. In many developing countries, limited technical capacity, inadequate monitoring, insufficient regulatory infrastructure, and lack of political will continue to weaken its implementation.

### Transboundary Shift of PFAS and PFAS-Containing Product Manufacture

One serious implication of the new or proposed PFAS regulations is that industries might exploit weak or absent PFAS-relevant regulatory systems in developing countries by relocating manufacturing units involving PFASs to these regions. For instance, current market trends show that the fluoropolymer market in Africa is expected to grow at a compound annual growth rate of about 9%, increasing from USD 7.2 million in 2024 to around USD 11 million in 2029. Interestingly, several key companies with a strong market presence and business interests in both the EU and the US are contributing to this growth.^42^ A similar trend has earlier been observed in some Asian countries, including China, where a noticeable rise in the use and production of certain PFASs, including replacements for PFOS and PFOA, has been reported.^43^ Notably, this also coincides with the period from 2000 to 2015 when 3M, Chemours/Dupont, and other major PFAS manufacturers (companies that participated in the USEPA’s PFOA Stewardship Program) ceased production of some well-known PFASs, including long-chain perfluoroalkyl acids and their precursors, due to regulatory pressures in the EU and the US. Given the competitive drive for economic growth in Asia, many Southeast Asian countries may follow a similar trajectory in the absence of strict PFAS restrictions along with changes in the global business dynamics of PFAS-based industries. In certain cases, PFAS-producing facilities that ceased to operate in developed countries have already transferred their technology, equipment, and market to chemical companies operating in developing countries. A recent example is the case of Italy’s Miteni factory, which produced PFASs and contaminated major water resources in the Veneto region of Italy. After facing legal proceedings in Italy, Miteni sold its machinery and patents, including those required for PFAS production, to Viva Lifesciences, a subsidiary of the Indian chemical company Laxmi Organic Industries. Laxmi has already built a factory in a previously pristine area of Maharashtra, in Lote, and intends to produce PFASs, including fluorochemicals compatible with Miteni’s product list, and capture the market share formerly held by Miteni.^44^ While major PFAS manufacturers still remain concentrated in countries and regions such as the USA, Europe, China, and Japan, available PFAS inventories and environmental contamination data indicate a growing influx of PFAS-intensive industries (e.g., fluoropolymer production, electroplating, TULAC, and lithium-ion batteries) into the markets and industrial spaces of developing countries. This trend is often obscured by the banners of economic development while carrying the risk of creating new PFAS pollution hotspots in developing countries.^45–47^ For instance, the government of India has recently approved a major investment (USD 15.2 billion) to boost domestic semiconductor manufacturing as well as attracting foreign firms to set up operations in the country.^48^ Similarly, an assessment carried out by the World Bank showed a strong economic case for accelerating electric vehicles (EVs) adoption in 20 selected low- and middle-income countries.^49^ Some of these countries, such as India, are rapidly progressing toward self-sustaining EV production,^50^ which still seems to be a PFAS-intense sector despite the increase of rigorous and successful efforts to develop PFAS-free alternatives.^22^

The disparity in PFAS regulations and the resulting transboundary shift of manufacturing also means that industries can continue to produce and use certain groups of PFASs in countries with weak and outdated regulations on occupational exposure and environmental emissions of PFASs and chemical pollutants in general. The products from these countries and their manufacturers would still be suitable for export to developed countries with stricter PFAS restrictions, for instance via mail-order consumer products, all while remaining inexpensive. This issue particularly extends to industries with heavy PFAS usage, such as TULAC, packaging, and electronic manufacturing. In such cases, PFAS pollution would not remain confined to certain regions such as in Asia (where a lot of clothing and electronic manufacturing takes place) but could be recirculated globally. This concern has recently been underscored based on the latest updated dossier of the EU’s PFAS restriction proposal, which allows producers to continue exporting PFASs to non-EEA countries indefinitely, regardless of what they will be used for.^51^ This not only shifts PFAS burdens and risks abroad but also keeps transboundary PFAS movement and exposure pathways to EU and non-EU populations active.

### Relocation of PFAS Hotspots and Recirculation of PFAS Pollution

Both the trade of PFAS stocks, PFAS-containing products, and associated wastes, as well as the transboundary shift in the manufacturing of PFAS and PFAS-containing products, will contribute to the emergence of new PFAS pollution hotspots in developing countries. Additionally, growing consumerism and evolving material-intensive lifestyles in developing countries are expected to further drive an increased demand for and consumption of PFAS-containing products, such as packaged food, cosmetics, consumer goods, and electronics (including EVs). While consumer products may not themselves be the primary sources of PFAS emissions, their growing demand and consumption is linked to the expansion of downstream PFAS-intense industries such as TULAC, fluoropolymer, and lithium-ion battery manufacturing.^52^ These industries are already recognized as dominant PFAS emission sources, especially in countries where regulatory oversight and effectiveness are compromised.^45^ As a result, PFASs, which were previously unobserved in these regions or present at lower concentrations, could be found at levels similar to those in developed countries. This relocation of PFAS hotspots would ultimately contribute to the global recirculation of PFASs. Such recirculation can occur through market pathways, such as contaminated food and seafood from these regions being exported. It can also occur through environmental pathways, since many PFASs can undergo long-range atmospheric and oceanic transport. For example, volatile PFAS precursors, such as fluorotelomer alcohols (FTOHs), can travel considerable distances before degrading into stable compounds like perfluoroalkyl carboxylic acids (PFCAs).^53^ Such recirculation of toxic chemicals has already been reported for various banned endocrine-disrupting chemicals (including some PFASs), when food and feed imported to the EU from developing countries were found contaminated with high levels of these chemicals.^54,55^

### Increasing Pressure on Tackling Other Critical Environmental Challenges in Developing Countries

Recent studies have shown that the cost of PFAS pollution, both in terms of its health and environmental impacts, is enormous. For instance, the burden of plastic-attributable diseases caused solely by PFAS exposure in the US in 2018 was estimated to be 22 billion USD.^56^ A similar assessment for Europe estimated that annual health-related costs associated with PFAS exposure range between 52 and 84 billion EUR for all EEA countries.^29^ Moreover, the societal cost of PFAS pollution—which includes the costs of soil and water remediation, healthcare, and biomonitoring—is estimated to be about 3 orders of magnitude higher than health-related costs alone. A recent assessment published by the European Commission in 2026 found that full compliance with its Environmental Quality Standards for PFASs would cost up to €1.7 trillion by 2050.^200^ An upper estimate is that the global societal cost of PFAS pollution could reach up to about $17 trillion annually if no PFAS restrictions are implemented.^57^ Even if the uncertainty of such estimates is considered, it is clear that the cost associated with PFAS pollution would be unaffordable for developing (and many developed) countries. Developing countries already lack modern infrastructure and capacity to manage PFASs along with other toxic chemicals (especially from their drinking water and soils) and would thereby require substantial investment for upgrading. While outdated infrastructure is also a concern in developed countries due to heavy costs of replacement (e.g., aging drinking water systems in the USA),^58^ the situation is considerably more critical in developing countries. For instance, in India the coverage of wastewater treatment plants is inadequate both on a per-capita basis and relative to river catchment areas.^59,60^ Furthermore, most water treatment plants in developing countries still rely on conventional treatment technologies, which are mostly incapable of removing many PFASs, particularly short-chain and ultrashort-chain PFCAs, as compared to modern hybrid treatment systems that can remove a larger spectrum of PFASs.^61,62^ Similarly, hazardous (municipal and industrial) waste management systems are either absent, have insufficient capacity, or are underdeveloped, resulting in inadequately treated waste and sewage sludge (biosolids) being released into surface waters or applied to agricultural soils, which is an issue in both developed and developing countries.^61,63,64^ This disparity means that while developed countries face challenges in upgrading or retrofitting their existing infrastructure, developing countries often must build their infrastructure almost from scratch, leading to a much larger financial burden on them in terms of PFASs (and other chemicals) management. Given the very limited budget (and infrastructure) available for overall environmental issues in developing countries, newly relocated PFAS hotspots to these countries would compromise management efforts dedicated to basic environmental and sustainable development issues related to climate change and urbanization, such as PM2.5 air pollution, water scarcity, diminished biodiversity/ecosystem services and increased multistressors to human health.

## Immediate Actions to Address Global Disparities in the Regulations of PFASs

Implications of these critical issues arising from the disparities in PFAS regulations globally can be minimized through a series of immediate actions described below.

### Inclusion of PFASs in Regulations in Developing Countries

The EU and many other developed countries took more than a decade to develop regulatory mechanisms to manage PFASs. These regulations are based on extensive research and intense negotiations between policymakers and industries. Developing countries should utilize the experience (from both research and negotiations) of the developed countries and timely develop adequate regulatory mechanisms in coherence with their domestic political scenarios to effectively manage PFASs. Currently, many developing countries including India and Bangladesh, which are also hubs of industries with intensive PFAS use, such as the TULAC sector, lack clear regulations to ban or restrict PFAS use, production, and trade.^17^ A recent assessment of regulations in Asian and Middle Eastern countries found that only a few have in-place regulatory mechanisms to manage only a small subset of PFASs.^17^

Importantly, beyond the absence of PFAS-specific regulations, many developing countries also lack overarching and comprehensive national chemical regulations, such as those in the EU (i.e., REACH), which cover a large range of industrial chemicals and set strict rules for their use, trade, and manufacturing.^65,66^ In the absence of such comprehensive regulation, each new toxic chemical or group of chemicals requires fragmented negotiations and regulatory processes that must be initiated from the very beginning. This approach ultimately delays necessary actions and allows pollution to escalate. Furthermore, this gap leaves governments without regulatory mechanisms and institutional authorities to systematically address immediate pollution threats such as PFASs, or for that matter other emerging contaminants in the future. On the other hand, developed countries with comprehensive chemical regulations can more efficiently integrate restrictions on PFASs (or other emerging contaminants) into their existing frameworks.

Many developing countries, including India, are anticipated to be hotspots of PFAS pollution;^67^ however, beyond the absence of comprehensive chemicals regulation, they face additional institutional and policy gaps in addressing individual sets of chemicals, including the small subset of PFASs listed under the Stockholm Convention. As of now, India and many other developing countries are yet to ratify the Stockholm Convention’s updates on selected PFASs.^17,68^ While some developed countries, such as Australia and the USA, are not bound by these specific listings of PFASs under the Convention, because they either have not ratified these amendments or are not party to the Convention, they have nevertheless implemented local and national regulations that restrict a larger number of PFASs than those currently listed under the Convention (see Table 1). Bringing comprehensive domestic PFAS-related regulations (along with overarching national chemical regulations) into existence in developing countries would accelerate the management of PFAS pollution (and other emerging chemical pollution threats in the future) locally and globally. Such efforts would also support these countries in more effectively fulfilling their commitments under various international chemical management agreements, including those under the Stockholm Convention.

Overall, PFAS management must be addressed through both stronger national regulations and robust implementation of relevant multilateral environmental agreements. These together control two distinct dynamics that emerge from regulatory disparities between developed and developing countries. On the one hand, stronger national regulations minimize the shift of PFAS production and use in developing countries (a “race to bottom”), which is not illegal but nevertheless exploits regulatory asymmetries and shifts environmental and health burdens to disadvantaged populations. On the other hand, strict implementation of multilateral environmental agreements is needed to prevent illegal trade or uncontrolled transboundary movements of hazardous wastes. Controlling both these dynamics is also relevant to keep a strict check on expansion of PFAS-intense industry in less regulated markets and to act against unlawful trade and dumping.

### Increasing International Funding for Monitoring and Reducing Emissions of PFASs

Regular monitoring of PFASs in the environment and human population is an important prerequisite for effectively managing PFAS emissions and associated health risks, as well as to validate that the policies focused on substance bans or pollution prevention are having an impact. Experience with other harmful and persistent chemicals (e.g., CFCs) reflects that globally coordinated monitoring is crucial for identifying major emission sources as well as illegal transboundary movement, trade, and use.^38,39^ The effectiveness and precision of such monitoring in detecting illegal point sources of emissions, use, and production of banned/restricted substances depends on the spatial resolution and coverage of monitoring stations, particularly in developing countries, where the risk of bypassing a law is relatively higher.^40,41^ PFAS monitoring and overall PFAS research have been insufficient in many developing countries.^69^ These countries lack networks of environmental and human biomonitoring of PFASs. Apart from the research on PFAS exposure and risk to the environment and humans, a dedicated segment of research should focus on systematically mapping PFAS stocks, uses, and sources in the specific socioeconomic and cultural contexts of these developing countries. The composition of their PFAS uses and sources might significantly differ from that of developed countries due to considerable differences in lifestyle, consumer choices, and economic priorities, and thus may require adjustments in regulations and policies concerning PFASs.

Developed countries (particularly those in Europe and North America) have made substantial financial investments over the years to carry out extensive and systematic PFAS monitoring. For example, this monitoring is carried out through the Human Biomonitoring for Europe (HBM4 EU) project in the EU (the 27 EU member states plus Norway, Switzerland, Iceland, and Israel) and through National Health and Nutrition Examination Survey (NHANES) in the US.^70,71^ Researchers and think tanks are now recommending increased spending on research to find safe PFAS alternatives as well as on remediating existing PFAS contaminated sites and drinking water.^22,72^ Spending on PFAS monitoring (or even overall chemical pollution) and research is low in developing countries, as reflected by the limited amount of research outcomes on PFASs originating from these countries (with the notable exception of China, which is a major contributor to global research on PFASs).^69^ While strengthening PFAS monitoring networks is essential to identify PFAS release and exposure sources and to detect illegal activities, developing countries must also invest heavily in preventive measures to reduce PFAS pollution at the source. These measures may include adopting safer alternatives to PFASs where available,^23,24^ strengthening import controls for PFAS-intense products,^73^ establishing industry-specific guidelines and/or aid for phasing out PFAS uses, tightening industrial emission regulations toward zero emissions of all PFASs, building capacity for municipal and industrial waste treatment and management, and fostering technology transfer and capacity building through international cooperation (Table 2). Considering the resource limitations in developing countries, a balanced approach combining both monitoring and prevention will be necessary to effectively manage PFASs, where monitoring provides an evidence-base to guide actions and prevention measures.

**Table 2:** An Overview of Immediate Steps for Managing PFAS Pollution in Developing Countries

Inclusion of PFAS ban and restrictions in domestic regulations	Systematic PFAS monitoring and prevention	Co-creation by partnering with global and private sectors
Adapt from relevant regulations in developed countries	Regular monitoring with adequate spatial and temporal coverage	Build capacity with global and private partnerships on PFAS monitoring and preventions (Overcome resources challenges: funding, capacity, expertise, etc.)
Set and adopt limits for environmental and human exposure to PFASs	Expand existing environmental health surveillance systems to cover PFASs and other priority chemicals	Utilize the Intergovernmental Science-Policy Panel on Chemicals, Waste and Pollution to bridge gaps between developed and developing countries
Strict compliance with multilateral environmental agreements	Create interoperable monitoring and data collection platforms (FAIR data principles)	Explore ways of implementing and utilizing the “polluter pays principle”
	Leverage and extend global and regional initiatives (Norman Network, IPCHEM, HBM4 EU, US-NHANES, CompTox, GAPS network, Ozone monitoring)	Aid and compel industries to phaseout PFASs
	Promote adoption of PFAS alternatives and support research on them	Establish regional collaborations, shared laboratories, protocols, and pooled databases.

To ensure that new investments in PFAS pollution monitoring and prevention in developing countries generate maximum impact, it is crucial to design programs that are interoperable with existing (or upcoming) global initiatives and adhere to principles of “findable, accessible, interoperable, and reusable” (FAIR) data. Data platforms that are common repositories for environmental and health monitoring data are a precondition for exploiting the full potential of scientific data in policy making. Several existing global programs already demonstrate that interoperable systems (based on the FAIR principles) can guide both effective chemical pollution monitoring and prevention. For instance, the Norman Network has shown the benefits of harmonized monitoring and collaborative data sharing across Europe. Similarly, the EU’s proposed common chemical data platform, which builds upon the existing database of IPCHEM and ECHA, illustrates the importance of centralized data repositories in supporting comprehensive risk assessment and regulatory actions on chemicals. Further lessons can also be drawn from the USEPA’s CompTox Chemical Dashboard, which includes numerous data on PFASs. This highlights the power of a centralized, accessible platform for chemical data (chemistry, toxicity, and exposure) to support regulatory decisions, the importance of integrating diverse data sources and predictive models, and the need for reliable data quality and curation.^74^ Extending such networks to include contributions from developing countries would improve global PFAS risk assessments and strengthen these countries’ ability to comply with multilateral environmental agreements. At the same time, this will also benefit developing countries in avoiding substantial economic costs and scientific labor in setting up such platforms from scratch (Table 2).

To build their own capacities for monitoring and preventing PFAS exposure and risks, developing countries can also draw lessons from large-scale environmental and human biomonitoring initiatives such as the HBM4 EU, the US NHANES, and air, water, and soil contamination monitoring networks coordinated under the European Monitoring and Evaluation Programme (EMEP). Implications of these programs go beyond just tracking the problem. Since these programs are generally interoperable and based on the FAIR data principles, their monitoring data provide the crucial evidence base needed for identifying priority emission sources, exposure hotspots, and vulnerable populations, and thereby enable effective policymaking and targeted interventions preventing chemical pollution. However, building such comprehensive and systematic programs in developing countries would require large financial resources. At the beginning, one way to tackle this is by integrating PFAS monitoring into existing water quality, food safety, and public health surveillance systems. This will make it possible to leverage existing infrastructure. This can be further complemented by regional collaborations including shared laboratories, protocols, and pooled databases across countries. To further ensure that developing countries build scientifically credible and resilient PFAS (and other chemicals) monitoring and prevention systems, their domestic efforts should be further supported by developed countries, including by transferring technology, building capacity, and targeted funding under bilateral or multilateral environmental agreements (Table 2).

Developing countries can extract specific, locally appropriate lessons from established global monitoring initiatives under multilateral environmental agreements, for example, the global monitoring plan (GMP) of the Stockholm Convention,^75–77^ worldwide air monitoring of ozone-depleting chemicals,^78^ and the global mercury observation system under the Minamata Convention.^79^ The GMP of the Stockholm Convention establishes a coordinated framework for the systematic collection of comparable monitoring data on legacy POPs and POP–PFASs, including PFOS, PFHxS, PFOA, and long-chain PFCAs across all UN regions.^80^ These programs have clearly shown that standardized and interoperable monitoring protocols, centralized data sharing platforms, and sustained funding are crucial for global actions to minimize chemical pollution. Similarly, the Global Atmospheric Passive Sampling (GAPS) network, which monitors selected PFASs and other POPs worldwide, has demonstrated that even cost-effective monitoring techniques (e.g., passive sampling) along with collaborative networks are capable of guiding policy actions and effectiveness evaluations even for regions with limited infrastructure.^76,81^

Further, to better outline the scale of the PFAS pollution problem and support effective decision making for PFAS management in developing countries, both the government and private sector must enhance funding, primarily for PFAS monitoring and building capacity and infrastructure for remediating PFAS contamination (in drinking water, wastewater, and soil), and in parallel for finding safe and locally adoptable alternatives to PFASs. Current international collaborations of national research councils, such as the Global Research Council (GRC) (https://globalresearchcouncil.org/) could be well suited to fund and administrate such research, to ensure participation and cocreation from developing countries.

### Co-Creation with Developed Countries

PFASs and chemical pollution in general is a global problem that requires coherent and collective management efforts at the global scale, rather than fragmented regional efforts. Leaving behind developing countries in chemical management actions will have repercussions in the long term for developed countries as well, particularly considering that PFASs are highly persistent and able to move over boundaries through air, water, food, and feed, even to remote locations where they were not previously produced or used. Developed countries can help boost PFAS management initiatives in developing countries by cocreating solutions with them. This would include sharing relevant knowledge and infrastructure and through dedicated high-level workshops and events with industry and policy makers from both developed and developing regions, financial support, and more meaningfully by cocreation in which the developed and developing countries work together through a mandate of PFAS reduction and replacement. This should include interventions to upgrade PFAS research infrastructure in developing countries to develop and implement state-of-the-art analytical methods, instrumentation as well as investing in safe and sustainable innovation. Such a mandate of cocreation for PFAS solutions could also be considered within the auspices of the “Intergovernmental Science-Policy Panel on Chemicals, Waste and Pollution”, based on resolution 5/8 of the United Nations Environment Assembly.^82^

Co-creation also presents a crucial opportunity for the developed world, particularly on the basis of the Polluter Pays Principle (PPP),^83^ given that a significant part of PFAS pollution in developing countries originates from industrial activities, products, and waste streams linked to developed nations. Under the PPP, developed countries and the corporations from these countries have a defined responsibility to support chemical pollution mitigation efforts in the regions most affected by their pollution. In order to implement the PPP, financial mechanisms and instruments for polluting industries, such as taxation or fees based on PFAS use or emissions, need to be embedded in policy, and administered by (inter)­government entities. The revenue thus collected could be invested in initiatives to share expertise and develop research infrastructure, in capacity building, and implementing long-term PFAS reduction strategies in developing countries. By embracing cocreation under PPP mechanisms, developed countries can also move beyond fragmented, short-term interventions and instead drive systematic, equitable solutions that address the global issue of PFAS pollution while ensuring accountability for their global PFAS footprints.

Developing countries should leverage modern guidelines and methods for safe chemical management, including those related to PFASs. Specifically, areas where knowledge can be leveraged include interventions for safely handling hazardous chemicals, evaluating the costs and benefits of PFAS management in various socioeconomic scenarios, developing safer alternatives for PFASs or PFAS-containing products, and integrating chemical management into the core safety and sustainability management systems of local industries.

On the regulatory front, developed countries, through their modern chemical management regulation must ensure that incidences of illegal exports of banned pesticides, hazardous chemicals, and e-waste from these countries are not repeated for discarded PFASs and PFAS-containing products. This will help to prevent the exploitation of weak and vulnerable chemical regulations in developing countries. PFAS restrictions in developed countries must also ensure that manufacturers do not just relocate the production of PFAS-containing products to developing countries.

## Space for a More Cohesive Global Effort

Considering the scale of the PFAS pollution problem, isolated regional efforts, though valuable, will not be sufficient to manage a global problem of this nature. If regulatory gaps in PFAS pollution management persist, these regional actions risk shifting the PFAS burden to developing countries as well as minimizing the impact on global PFAS emissions. Over the last two decades, multiple global and regional initiatives have made crucial contributions to understanding the occurrence of PFASs and managing their associated risks. For example, the Stockholm Convention has banned or restricted a small subset of PFASs. The OECD has played an important role in characterizing and understanding PFASs, estimating their production and emissions, and synthesizing information on available safer alternatives to PFASs. The WHO is developing guideline values for PFOS and PFOA in drinking water.^84^ Furthermore, academic-media collaborations, such as the Forever Pollution Project led by European journalists in collaboration with international academics, including via the Global PFAS Science Panel network, identified nearly 23000 PFAS-contaminated sites across Europe.^85^ However, the coverage of these initiatives and their progress still remains uneven across regions, and PFAS pollution continues to expand, including in developing countries.^86,87^ In this context, the newly established Intergovernmental Science-Policy Panel on Chemicals, Waste and Pollution can be viewed as an opportunity that can help mitigate the regulatory as well as knowledge gaps between the developed and developing worlds by integrating scientific knowledge, bridging national efforts, and strengthening coordination across the existing and upcoming actions on PFASs.^82^ Policymakers and researchers involved in the regional regulatory development concerning PFASs should actively contribute to these global processes to prevent shifting the PFAS problem elsewhere and to accelerate the collective reduction of PFAS pollution globally. It is high time that the governments, researchers, and private sector in developing countries come together to take steps to systematically monitor, map, and reduce the import, use, and emissions of PFASs, and to build solid support for the relevant local regulatory developments.
